# Identification and systematic annotation of tissue-specific differentially methylated regions using the Illumina 450k array

**DOI:** 10.1186/1756-8935-6-26

**Published:** 2013-08-06

**Authors:** Roderick C Slieker, Steffan D Bos, Jelle J Goeman, Judith VMG Bovée, Rudolf P Talens, Ruud van der Breggen, H Eka D Suchiman, Eric-Wubbo Lameijer, Hein Putter, Erik B van den Akker, Yanju Zhang, J Wouter Jukema, P Eline Slagboom, Ingrid Meulenbelt, Bastiaan T Heijmans

**Affiliations:** 1 Molecular Epidemiology, Leiden University Medical Center, Leiden, The Netherlands; 2 Netherlands Consortium for Healthy Aging, PO Box 9600, Leiden 2300, RC, The Netherlands; 3 Medical Statistics, Leiden University Medical Center, Leiden, The Netherlands; 4 Department of Pathology, Leiden University Medical Center, Leiden, The Netherlands; 5 The Delft Bioinformatics Lab, Delft University of Technology, Delft, The Netherlands; 6 Department of Cardiology, Leiden University Medical Center, Leiden, The Netherlands

**Keywords:** Differentially methylated region, Illumina 450k, Annotation, Algorithm, Tissue

## Abstract

**Background:**

DNA methylation has been recognized as a key mechanism in cell differentiation. Various studies have compared tissues to characterize epigenetically regulated genomic regions, but due to differences in study design and focus there still is no consensus as to the annotation of genomic regions predominantly involved in tissue-specific methylation. We used a new algorithm to identify and annotate tissue-specific differentially methylated regions (tDMRs) from Illumina 450k chip data for four peripheral tissues (blood, saliva, buccal swabs and hair follicles) and six internal tissues (liver, muscle, pancreas, subcutaneous fat, omentum and spleen with matched blood samples).

**Results:**

The majority of tDMRs, in both relative and absolute terms, occurred in CpG-poor regions. Further analysis revealed that these regions were associated with alternative transcription events (alternative first exons, mutually exclusive exons and cassette exons). Only a minority of tDMRs mapped to gene-body CpG islands (13%) or CpG islands shores (25%) suggesting a less prominent role for these regions than indicated previously. Implementation of ENCODE annotations showed enrichment of tDMRs in DNase hypersensitive sites and transcription factor binding sites. Despite the predominance of tissue differences, inter-individual differences in DNA methylation in internal tissues were correlated with those for blood for a subset of CpG sites in a locus- and tissue-specific manner.

**Conclusions:**

We conclude that tDMRs preferentially occur in CpG-poor regions and are associated with alternative transcription. Furthermore, our data suggest the utility of creating an atlas cataloguing variably methylated regions in internal tissues that correlate to DNA methylation measured in easy accessible peripheral tissues.

## Background

Epigenetic mechanisms, including DNA methylation, are essential in mammalian development and cell differentiation [1]. Several studies have compared genome-wide DNA methylation patterns, particularly of cytosine at CpG dinucleotides, between human cell types and tissues to identify general characteristics of genomic regions that define epigenetic differences between tissues [2-4]. However, these studies often focused on a subset of regions either because of *a priori* hypotheses or due to the limited coverage of the DNA methylation profiling technology used. For example, while many studies have explored and identified tissue-specific differentially methylated regions (tDMRs) at promoter sequences [2,4-8], differential methylation at other genomic regions has been investigated less widely and consistently. Several studies focussed on CpG islands (CGIs), which are genomic regions with a high density of CpGs, and reported the predominant occurrence of tDMR CGIs located in the gene bodies [9-11] and described their potential role in regulating alternative transcription start sites [10]. One study highlighted the 2 kb region flanking CGIs (that is, CGI shores) as a frequent target of tissue-specific methylation [12], but this finding was not replicated in a mouse study [9].

To study the contribution of epigenetic variation to human disease risk, it is necessary not only to study tissue differences, but also to explore the correlation of DNA methylation signatures between tissues. Many diseases involve internal organs (IOs) that cannot be sampled in human subjects participating in epidemiological studies. Studies of such diseases would be facilitated if methylation of DNA from peripheral tissues could be used as a proxy; that is, if inter-individual variation in DNA methylation levels at a genomic region that is observed in a population is positively correlated with that in an (unmeasured) internal organ. Although candidate region [13] and genome-wide [11] studies suggested that correlated DNA methylation across tissues may occur, little is known about the prevalence of such correlations.

In this study, we explored genome-wide DNA methylation in six internal and four peripheral tissues in two independent datasets using the Illumina 450k methylation chip [14,15]. Apart from systematically covering promoter regions, CGIs and CGI shores, the chip targets sufficient CpG dinucleotides outside these regions to study other annotations. We implemented an algorithm to identify tDMRs, which allowed us to detect statistically robust and biologically relevant tDMRs in 450k data. This allowed us to evaluate previously indicated annotations of tDMRs systematically in a single study. In addition, we explored annotations utilizing more recent insights on genome biology including those from the ENCODE project. Finally, we evaluated the occurrence of correlated DNA methylation across tissues.

## Results

### Identification of tDMRs

Genome-wide DNA methylation data was generated from four peripheral tissues (blood, saliva, hair follicles and buccal swabs) from five individuals, and six internal tissues (subcutaneous fat, omentum, muscle, liver, spleen and pancreas) and blood from six individuals, using Illumina 450k DNA methylation chips (Additional file 1: Table S1). The DNA methylation patterns observed in the tissues were in concordance with previously described characteristics: the distribution of DNA methylation was bimodal with a minority of CG dinucleotides showing intermediate DNA methylation levels (Additional file 2: Figure S1A, B); the canonical pattern of low DNA methylation around transcription start sites (TSSs) was observed (Additional file 3: Figure S2A); and, finally, adjacent CpGs within 1 kb had similar DNA methylation levels (Additional file 3: Figure S2B).

**Figure 1:**
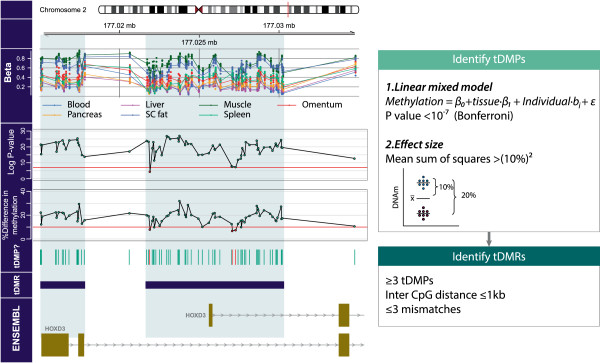
**Example of the tDMR finder algorithm used for the *HOXD3 *gene.** Tissue-specific differentially methylated regions were identified in a two-step approach: first, we identified tDMPs. CpGs were considered to be tDMPs when there was a genome-wide significant mean difference of ≥ 10%. The mean difference was expressed as a mean sum of squares. A difference ≥ 10% equals a mean sum of squares ≥ 0.01 (square of 10% = 0.1^2^). To test whether the difference was significant, we applied a linear model per CpG site, with a random effect for each individual to correct for any inter-individual variation. From this linear model we obtained a *P* value (F-test) per CpG site and used a multiple testing corrected *P* value as a cut-off (10^-7^). Second, we identified tDMRs as regions with at least three tDMPs with an inter-CpG distance of at most 1 kb and a maximum of three non-tDMPs. Mb, megabase; tDMP, tissue-specific differentially methylated position; tDMR, tissue-specific differentially methylated region.

Tissue types tended to cluster together according to genome-wide DNA methylation data indicating the occurrence of tissue-specific methylation patterns (Additional file 2: Figure S1E, F). To study these patterns in more detail, we developed an algorithm to identify tissue-specific differentially methylated regions systematically using 450k methylation data as described in Figure 1 (also see Methods). Briefly, first tissue-specific differentially methylated positions (tDMPs) were identified. tDMPs were defined as CpGs with a DNA methylation difference between tissues that was: (1) genome-wide significant (*P* < 10^-7^) and (2) had a mean sum of squares ≥ 0.01 (equals (10%)^2^, that is, the mean of the difference between the individual tissues and the overall mean across tissues should be greater than 10%). Next, differentially methylated regions (DMRs) were identified as regions with at least three differentially methylated positions (DMPs) with an inter-CpG distance ≤ 1 kb, interrupted by at most three non-DMPs across the whole DMR (see Methods; the algorithm is in Additional file 4). The algorithm detected 3,533 and 5,382 tDMRs in the peripheral and internal tissue datasets, respectively (Table 1 and Additional file 5: Table S2). There were 4,877 unique (that is, non-overlapping) tDMRs between datasets. Interestingly, 2,019 tDMRs were detected in both peripheral and internal tissues (9,388 CpGs in common, *P* < 0.001). The tDMR distribution over the genome was similar for the two datasets (Additional file 3: Figure S2C). A further indication of the validity of the tDMRs was obtained from a visualization of the tDMRs in a heat map according to tissue, which showed the expected clustering by germ layer and confirmed the previously reported cellular similarities between blood and saliva, and between hair and buccal swabs (Additional file 6: Figure S3) [16].

**Table 1:** Characteristics of identified tDMRs

	***Number of DMR***	***Number of CpGs***	***Mean length (SD)***	***Median length (IQR)***
Peripheral tissues	3533	17067	605 (578)	442 (219–835)
Internal tissues	5382	27992	700 (646)	530 (259/942)
Unique between datasets	4877	26285		
Shared between datasets	2019	9387		

### tDMRs accumulate near genes expressed in specific tissues

tDMRs were mapped to their nearest gene and the TiGER database was used to verify the expectation that these genes are preferentially expressed in investigated tissues [17]. This was indeed the case (Additional file 7: Figure S4A, Additional file 5: Table S2). For example, tDMRs in the internal tissue dataset mapped preferentially to liver-specific genes (odds ratio for internal organs OR_I_ = 5.01, *P* < 10^-5^). In contrast, this was not observed in the peripheral tissue dataset (odds ratio for peripheral tissues OR_P_ = 1.02, *P* = 0.13). Enrichment of the blood-specific expression of genes adjacent to identified tDMRs was observed in both datasets (OR_P_ = 2.42, *P* < 10^-5^; OR_I_ = 1.88, *P* < 10^-5^). Furthermore, tDMRs mapping to genes with tissue-specific expression were hypomethylated in the tissue in which the gene is preferentially expressed compared with other tissues. This is in line with an inverse relationship between DNA methylation and expression (Additional file 7: Figure S4B). Taken together, these analyses indicate that our algorithm detected a tDMR set that is not only statistically robust but also biologically relevant.

### tDMRs associate with specific genomic annotations

**Figure 2:**
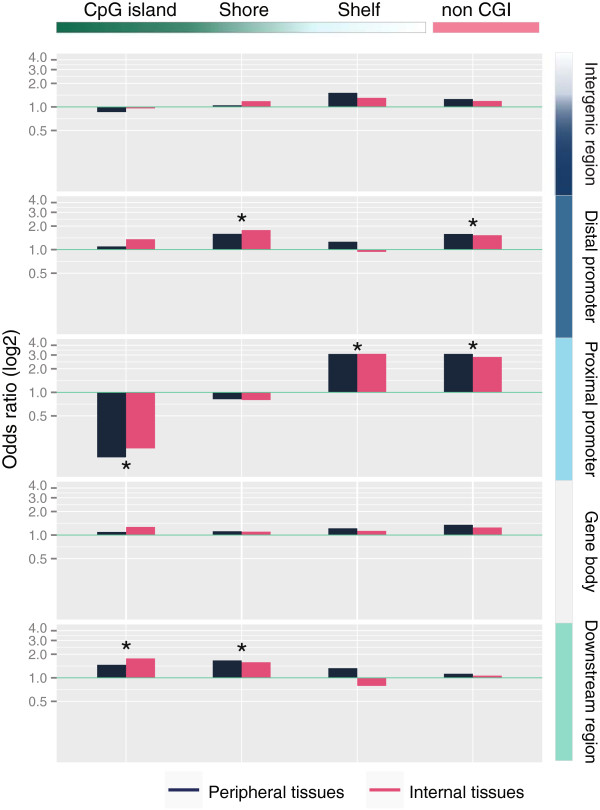
**Enrichment with tDMRs in the gene- and CpG-density centric annotation.** Differences were observed between CGI and non-CGI regions, especially in proximal promoters and downstream regions. Shores in distal promoters and downstream regions were enriched with tDMR CpGs. Enrichment with tDMR CpGs in non-CGI features was limited to distal promoters and proximal promoters. * *P* < 10^-5^. CGI, CpG island; tDMR, tissue-specific differentially methylated region.

**Figure 3:**
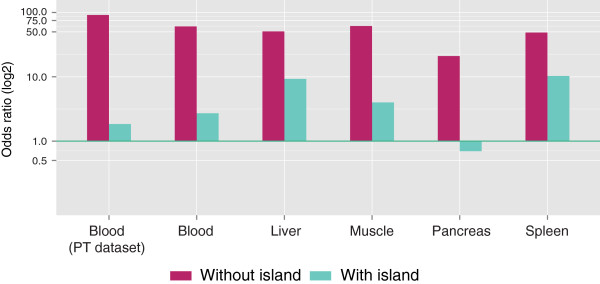
**Enrichment of tDMR CpGs in genes that are preferentially expressed in studied tissues.** Differential methylation of a non-CGI proximal promoter was strongly associated with tissue-specific expression (TiGER database [17]) of the adjacent gene and much more so than for differentially methylated CGI proximal promoters. tDMR CpGs were significantly enriched in all tissues in both proximal promoters with an island and proximal promoters without a CpG island (*P* < 10^-5^). PT, peripheral tissue; tDMR, tissue-specific differentially methylated region.

In order to systematically assess previous observations regarding tDMR annotations and to further explore annotations that became available more recently, we created extensive annotations of CpG sites interrogated with the 450k chip (the annotations can be found in Additional file 8) and evaluated their enrichment in tDMRs. First, tDMR CpGs were annotated according to the location relative to genes. This showed that the occurrence of tDMRs in proximal promoters (defined as −1500 to +500 from a TSS) was depleted, whereas it was enriched in other gene-centric annotations (Additional file 9: Figure S5). This pattern was highly concordant between internal and peripheral tissues (for example, for proximal promoters OR_P_ = 0.70 and OR_I_ = 0.68, *P* < 10^-5^). Next, we combined the gene-centric annotation with a CGI-centric annotation (Figure 2). The combined annotation revealed that the overall depletion in proximal promoters was due to a strong underrepresentation of tDMRs in CGI proximal promoters (Figure 2, OR_P_ = 0.15, OR_I_ = 0.19, *P* < 10^-5^). Conversely, non-CGI proximal promoters were strongly enriched for differential methylation (OR_P_ = 3.10, OR_I_ = 2.83, *P* < 10^-5^). Also in absolute terms, more tDMRs mapped to non-CGI proximal promoters (*n*_*P*_ = 781, *n*_*I*_ = 1,100) than CGI proximal promoters (*n*_*P*_ = 168, *n*_*I*_ = 313; Additional file 10: Table S3 and Additional file 5: Table S2). In proximal promoters, no enrichment of CGI shores was observed (OR_P_ = 0.82, OR_I_ = 0.80), while CGI shelves (that is, a 2 kb region flanking a CGI shore) showed a similar enrichment compared to the non-CGI proximal promoters (OR_P_ = 3.10, OR_I_ = 3.10, *P* < 10^-5^). In accordance with the preferential occurrence of tDMRs at non-CGI proximal promoters, the genes adjacent to these tDMRs were strongly enriched for tissue-specific gene expression, much more so than for CGI proximal promoters (Figure 3).

Other regions showing evidence for enrichment for tissue-specific methylation included CGIs in downstream regions (defined as the 3’ end to +5 kb relative to the 3’ end; OR_P_ = 1.46, *P* = 0.017; OR_I_ = 1.76, *P* < 10^-5^), CGI shores in distal promoters (OR_P_ = 1.59, OR_I_ = 1.78, *P* < 10^-5^) and CGI shores in downstream regions (OR_P_ = 1.67, *P* = 4 × 10^-4^; OR_I_ = 1.58, *P* = 1.2 × 10^-4^). Of note, no enrichment was observed for gene-body CGIs (defined as +500 kb to the 3’ end relative to the gene). Of the total number of tDMRs detected, ~25% overlapped with a CGI shore and a similar percentage with a CGI (Additional file 10: Table S3 and Additional file 5: Table S2). The number of tDMRs overlapping with CGI shelves was lower (~6%).

### tDMRs are enriched in alternative transcription start sites

**Figure 4:**
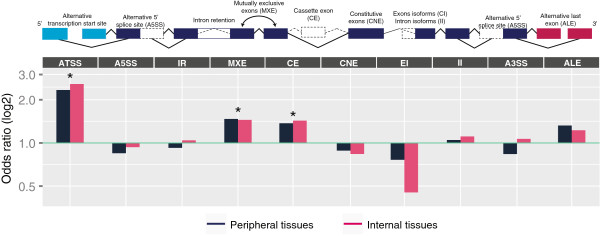
**Enrichment of alternative event regions with tDMR CpGs.** *P < 10^-5^. A3SS, alternative 3’ splice site; A5SS, alternative 5’ splice site; ALE, alternative last exon; ATSS, alternative transcription start site; CE, cassette exon; CNE, constitutive exon; EI, exon isoforms; II, intron isoforms; IR, intron retention; MXE, mutually exclusive exon; tDMR, tissue-specific differentially methylated region.

It has been suggested that DNA methylation regulates alternative transcription [18], which may be the mechanism underlying its contribution to tissue-specific expression. In support of this hypothesis, we observed enrichment of tDMRs in alternative transcription start sites (OR_P_ = 2.34, OR_I_ = 2.58, *P* < 10^-5^; an example is given in Additional file 11: Figure S6; see also Additional file 5: Table S2). This was also reflected in the number of tDMRs associated with alternative transcription start sites (PT: 18.8%, IO: 20.9%). In addition, significant enrichment was observed at mutually exclusive exons (OR_P_ = 1.47, OR_I_ = 1.45, *P* < 10^-5^) and cassette exons (OR_P_ = 1.37, OR_I_ = 1.43, *P* < 10^-5^) (Figure 4). Overall, 47.9% of tDMRs detected in the peripheral tissue dataset and 49.8% of the tDMRs detected in the internal organ dataset mapped to an alternative transcription event. It was previously indicated that methylation of CGIs primarily mediates the effects on alternative transcription [10]. We could replicate the presence of a tDMR at a CGI in the *SHANK3* gene body, which was found to regulate alternative transcription (Additional file 12: Figure S7) [10]. However, only a minority of tDMRs mapping to alternative transcription start sites (denoted by the occurrence of alternative first exons) were CGIs (*P*_*P*_ = 14.5%; *P*_*I*_ = 20.5%). The majority were non-CGI sequences (*P*_*P*_ = 52.5%; *P*_*I*_ = 48.3%) indicating a role for CpG-poor regions in the regulation of alternative transcription.

### Functional annotation of tDMRs

**Figure 5:**
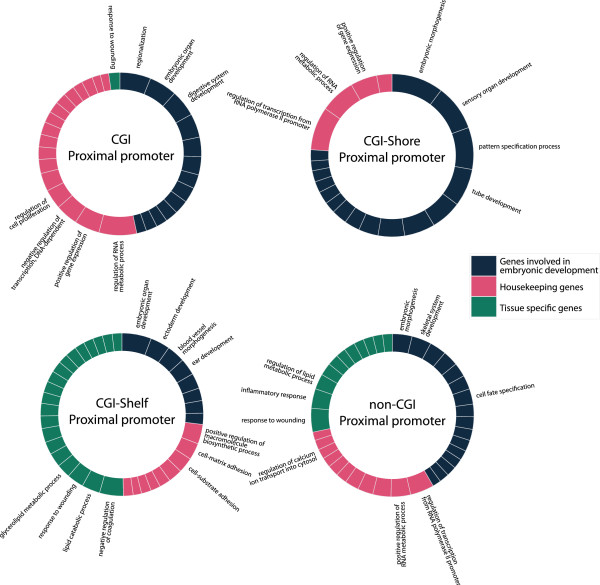
**Enrichment of GO terms with nearest genes of tDMRs.** Different colours represent the distinct major classes. Notice the difference in major classes between genes enriched with tDMRs that have a CGI or CGI flanking region and those which do not. When no CGI is present, tissue-specific genes are observed, while when there is a CGI present, the genes enriched with a tDMR are more often involved in embryonic developmental processes and gene regulation genes. Genes with a differentially methylated shelf overlapping with the proximal promoter, were associated with developmental -, housekeeping -, and tissue-specific GO terms. CGI, CpG island; GO, gene ontology; tDMR, tissue-specific differentially methylated region.

tDMRs were mapped to their nearest gene and enrichment analysis of gene ontology (GO) terms was used to describe functional categories. Non-CGI proximal promoters harbouring a tDMR were found to be involved in regulating tissue-specific processes reinforcing our previous observations of this class of tDMRs (Figure 5). In contrast, CGI proximal promoters harbouring a tDMR were largely associated with embryonic development processes. CGI shore proximal promoters with a tDMR were associated with similar processes as CGI proximal promoters with a tDMR, whereas CGI-shelf proximal promoters with a tDMR resembled non-CGI proximal promoters with a tDMR. The functional annotations of other tDMRs classes are given in Additional file 13: Figure S8.

### tDMRs are enriched for regulatory regions

Regulatory DNA is marked by DNase I hypersensitive sites (DHSs) [19]. DHSs were enriched for tDMRs (OR_P_ = 1.36, OR_I_ = 1.37, *P* < 10^-5^; Additional file 5: Table S2 and Additional file 14: Figure S9). Using ENCODE data on transcription factor binding sites (TFBSs) [20] we observed enrichment for tissue-specific methylation at the binding sites *BCL11A* (OR_P_ = 3.22, OR_I_ = 2.52, *P* < 10^-5^), *SUZ12* (OR_P_ = 1.71, OR_I_ = 2.17, *P* < 10^-5^) and *FOXA2* (OR_P_ = 1.12, *P* = 0.30; OR_I_ = 1.61, *P* < 10^-5^). Hypomethylation at TFBSs was observed in tissues in which the transcription factor is expressed (Additional file 15: Figure S10). For example, *FOXA2* is active in the liver [21], pancreas [22] and potentially hair follicles [23], and *FOXA2* binding sites were relatively hypomethylated in these tissues. tDMRs, however, were depleted for many other TFBSs, including for methylation-sensitive transcription factors *YY1* (OR_P_ = 0.23, OR_I_ = 0.25, *P* < 10^-5^), *Egr-1* (OR_P_ = 0.41, OR_I_ = 0.41, *P* < 10^-5^) and *NFkB* (OR_P_ = 0.44, OR_I_ = 0.41, *P* < 10^-5^).

### Correlation of inter-individual variation across tissues

**Figure 6:**
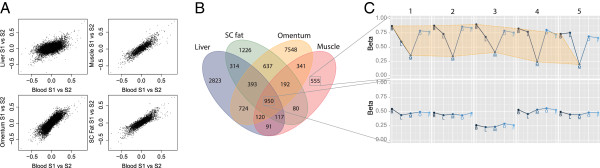
**Within-individual correlation in DNA methylation between tissues. (A)** Relation between differences within two individuals in blood versus one other tissue. **(B)** Venn diagram of the number of CpGs sites that are correlated between blood and one or more tissues. **(C)** Top: A variably methylated CpG site in muscle that is correlated with DNA methylation in blood. Bottom: A variably methylated CpG site that is correlated across all tissues likely due to the influence of SNPs. SC, subcutaneous; SNP, single nucleotide polymorphism.

We investigated the occurrence of inter-individual variation in the internal tissue dataset after exclusion of CpG sites overlapping with known SNPs. Although tissue-differences were the main driver of variation in DNA methylation, we observed inter-individual variation for 15,803, 11,719, 46,437 and 8,415 CpGs in the liver, subcutaneous fat, omentum and skeletal muscle, respectively (defined as a mean sum of squares > 0.025). The large number of variable CpGs observed in omentum may reflect the cellular heterogeneity of this tissue. For the variable CpG sites identified, we calculated the correlation between the between-individual difference for the internal tissue and the between-individual difference for blood (Figure 6A). When restricting these CpG sites to those with a correlation >0.8, the within-individual DNA methylation in blood correlated to variable DNA methylation in the liver, subcutaneous fat, omentum and skeletal muscle for 5,532, 3,909, 10,905 and 2,446 CpGs, respectively. Many of the correlated CpG sites were unique for a single internal tissue and blood but others were correlated across multiple tissues (Figure 6B). While the former may represent a genuine epigenetic correlation, in particular CpGs correlating across all tissues may frequently be driven by genetic variation influencing local DNA methylation (Figure 6C).

## Discussion

In this study we report on genome-wide methylation patterns generated using multiple peripheral and internal tissues from two independent sets of donors using 450k methylation chips. Although the 450k platform interrogates a small subset of the ~28M CpG sites in the human genome, it relatively comprehensively evaluates promoter regions and CpG islands, and also covers other potentially relevant features, including downstream genic and intergenic regions. A new algorithm was able to identify statistically robust tDMRs as illustrated by a statistically significant overlap in the location of tDMRs between the datasets. The biological relevance of the identified tDMRs was highlighted by the observation that they mapped to genes with tissue-specific expression and also showed hypomethylation specifically in the tissue expressing those genes. Annotation of tDMRs showed that they can occur irrespective of their position relative to genes or local CpG density. Tissue-specific DNA methylation was most evident, however, both absolutely and relatively, in regions outside CGIs or CGI flanking regions. This confirms previous studies reporting a high prevalence of CpG-poor regions near genes with tissue-specific expression both in humans [2,3,7] and animals [24,25].

One of our key findings is that the role of non-CGI tDMRs may frequently involve the regulation of alternative transcription. Tissue-specific methylation was associated with alternative transcription start sites and, despite being sparsely covered by the 450k chip, mutually exclusive exons and cassette exons. A previous study adopting a descriptive approach combined with functional validation suggested a primary role for DNA methylation at CGIs in alternative transcription [10]. Although we could confirm tissue-specific methylation at CGIs with a validated effect on alternative transcription from that study, our statistical approach highlighted the role of non-CGI regions in alternative transcription start sites. Interestingly, a recent study also supported a role for DNA methylation in controlling mutually exclusive exons underlining the validity of our results [26]. The link between DNA methylation, non-CGI sequences and alternative transcription arising from our data is in line with their hypothesized role in vertebrate evolution [27].

Recent studies of differential methylation between tissues emphasized the occurrence of tDMRs outside non-CGI and CGI proximal promoters. For example, studies of animal models [5,9] and subsequently humans underscored the occurrence of tDMRs in gene-body CGIs [11]. Although the 450k chip comprehensively assesses methylation at CGIs, only ~4% of the tDMRs detected in our study mapped to a gene-body CGI. Another feature that attracted significant attention is CGI shores, which are the 2 kb regions flanking CGIs. Irizarry *et al.* reported that 76% of the tDMRs identified overlapped with CGI shores [12]. Inspired by this work, the 450k chip was designed with the specific aim of covering CGI shores. Nevertheless, the percentage of CGI-shore tDMRs in our data was limited to ~25% of the total number of tDMRs. However, our data indicated that tissue-specific methylation at CGIs and CGI shores may be more relevant at downstream genic regions, which remain poorly studied. Of note, we found that differentially methylated CGI shores were associated with genes involved in housekeeping and developmental processes analogous to differentially methylated CGIs. tDMRs overlapping with so called CGI shelves (the regions flanking CGI shores) mapped to genes associated with tissue-specific processes, as was observed for non-CGI tDMRs. Our results indicate that the occurrence of tDMRs may be less biased towards previously suggested annotations including gene-body CGIs and CGI shores, and reinforce the potential utility of reconsidering current definitions of CGI annotations [12,28-30].

The annotation of tDMRs has thus far primarily focussed on CG content and location relative to genes. Increasing knowledge of genome biology can give a more in-depth annotation. The ENCODE project mapped DNase I hypersensitive sites (DHSs), informative markers of regulatory DNA and transcription factor binding sites (TFBSs) across 349 cell lines [19]. Both DHSs and TFBSs were enriched for tDMRs in our study. TFBS enrichment was observed for transcription factors (TFs) with a tissue-specific function and the TFBSs for these TFs were hypomethylated at TFBSs in the tissue in which they are expressed. These results are in accordance with the hypothesis that TF binding is associated with hypomethylation of TFBSs [31,32].

Although the largest variation in DNA methylation was observed between tissues, it is more relevant to investigate inter-individual variation from the perspective of epigenetic epidemiology, which aims at identifying epigenetic risk factors for disease. Epidemiological studies, however, often have to rely on accessible peripheral tissues as proxies for internal organs directly involved in the aetiology of the disease of interest [33]. Our exploration of the concordance between blood and internal tissues at CpG sites with variable DNA methylation suggested the presence of good correlations for a subset of variable CpG sites, many of which were locus and tissue-specific. Variable CpGs correlating across blood and all internal tissues may be primarily mediated by the effects of SNPs on DNA methylation [34] and may not necessarily represent a genuine epigenetic correlation. The initial evidence that blood DNA methylation may correlate to that of internal tissues as presented here and brain regions as reported previously [11] warrants investigations of more individuals and more tissues, such as the GTEx project [35], to work towards an atlas cataloguing those variably methylated regions in internal tissues that could potentially be studied indirectly by assessing their DNA methylation in specific peripheral tissues.

## Conclusions

In conclusion, using an effective approach to detect and annotate tDMRs in 450k methylation data, we highlight the importance of non-CGI regions in tissue-specific DNA methylation and provide further evidence for a role of differential DNA methylation in the regulation of alternative transcription. Moreover, our data suggest that peripheral tissues may to some extent be used to assess inter-individual differences in DNA methylation in internal organs that frequently remain inaccessible in epidemiological studies.

## Methods

### DNA isolation and Illumina 450k BeadChip

For the peripheral tissue dataset, five healthy volunteers from laboratory personnel (mean age 28 years, SD = 6.1) donated blood, saliva, hair and buccal swabs after providing informed consent. DNA was isolated from the blood using the Qiagen mini kit (Qiagen, Germany) using the manufacturer’s protocol. DNA from hair follicles was also isolated using Qiagen mini kits, with the addition of 3 μL dithiothreitol (DTT) during lysis to enhance the lysis of the hair follicles. DNA was isolated from saliva using Oragene Discover kits (OGR-250, DNA Genotek Inc). DNA from buccal swabs was isolated using a chloroform/isoamyl alcohol protocol [36]. For the internal tissue dataset, samples were taken from six cadavers within 12 h post-mortem (mean age 65.5 years, SD = 7.2; Additional file 1: Table S1). Blood was collected from the thoracic cavity in ethylenediamine-tetraacetic acid disodium salt dihydrate (EDTA) tubes (BD, United Kingdom). Tissue samples were collected and snap frozen onto a cork template with Tissue-Tek (Tissue-Tek, Netherlands). Samples were stored at −80°C until DNA extraction. To enhance lysis, tissues were sliced into 30-μm slices using a cryostat (Leica, Germany). For microscopic inspection, one 5-μm slice was stained with haematoxylin and eosin (HE). HE tissue slides were microscopically inspected to verify tissue integrity and homogeneity and to exclude inflammatory infiltrate. DNA was extracted using a chloroform/isoamyl alcohol protocol. DNA concentrations were determined using a PicoGreen dsDNA quantitation assay (Invitrogen). Bisulphite reactions were performed using the EZ-96 DNA methylation kit (Zymo Research, Orange County, USA) with an input of 1 μg of genomic DNA. After bisulphite conversion, each sample was whole-genome amplified, enzymatically fragmented, and hybridized to the Illumina HumanMethylation450 BeadChip.

### Ethics statement

This study was conducted according to the principles expressed in the Declaration of Helsinki. All samples were anonymized and procedures were performed according to the ethical guidelines in the Code for Proper Secondary Use of Human Tissue in The Netherlands (Dutch Federation of Medical Scientific Societies).

### (Pre-)processing of the Illumina 450k BeadChip data

All analyses were performed in using R statistics, version 2.15.1. SNPs on the array were used to confirm that tissue samples were from the same individual and CpGs on the X and Y chromosome were used to confirm gender. CpGs with a detection *P* value (a value representing the measured signal compared to negative controls) over 0.05 were removed from the data. Cluster analysis (based on Euclidian distance) did not reveal signs of batch effects. The distributions of the six different signals on the 450k array (Type I (red/green and methylated/unmethylated) and Type II (red/green)) were quantile normalized separately. Quality control plots were obtained using functions from the R package *minfi* and custom scripts [37].

### tDMR identification

Using the R package *IlluminaHumanMethylation450k.db*, Illumina identifiers were mapped to the hg19 genome build [38]. In order to objectively identify tDMRs we applied a newly developed algorithm (Figure 1). First differentially methylated positions were identified. The algorithm identifies tDMRs in two steps. CpGs were considered a tDMP on the basis of statistical significance and effect size. First we applied two linear models per CpG site, one with a fixed effect for tissue and one without (Figure 1):

(1) $$y_{j}=\beta_{0}+\beta_{1}\cdot T+b_{1}\cdot I+\epsilon$$

(2) $$y_{j}=\beta_{0}+b_{1}\cdot I+\epsilon$$

where *y*_*j*_ is the methylation value for CpG *j*, *β*_*1*_ the fixed effect for tissues and *b*_*1*_ is a random effect term for the individual. We tested whether the model with the fixed effect for tissue fitted the data better with the F test and used a Bonferroni corrected *P* value ≤ 10^-7^ (0.05/471k autosomal CpGs) as the threshold for statistical significance after correction for multiple testing. Statistical analysis was performed using the R package *lme4*[39]. Since individual CpG sites were evaluated, the statistical test was not influenced by the systematic difference between type 1 and type 2 probes on the 450k chip. Second, we calculated the measure for effect size and we used the mean sum of squares (analogous to the effect size parameter evaluated in the F test), which was calculated as:

(3) $$\frac{\sum {\left(\;{\bar{y}}_{i,j}-{\bar{y}}_{j}\right)}^{2}}{n}$$

where ${\bar{y}}_{i,j}\;$ is the mean methylation of tissue *i* of CpG *j*, ${\bar{y}}_{i}\;$ is the overall mean methylation of CpG *j* and *n* the number of tissues studied. The cut-off we used for the effect size was a 20% difference in DNA methylation between two tissues, which equals a ≥10% difference from the overall mean (≥10% difference equals a mean sum of squares ≥0.01 since the square of 10% = 0.1^2^). Using both an effect size and the *P* value cut-off, CpG sites were classified as tDMP or non-tDMP. In the second stage of the algorithm, we used the DMP status to identify DMRs, which were defined as ≥3 DMPs with an inter-CpG distance of ≤ 1 kb while allowing ≤ 3 non-DMPs in the complete DMR. This procedure assumes that the DNA methylation level of CpGs not measured using the 450k chip, but located in a tDMR called by the algorithm, are similar to the CpGs that were measured and led to the calling of a tDMR. This assumption is based on previous studies that reported high levels of co-methylation at shorter genomic distances (<1 kb) particularly in non-repeat regions (as interrogated using the 450k chip), for example, in candidate loci [13], in 27k data [34] and in whole genome bis-seq data [40]. The presence of co-methylation was confirmed in the current dataset (Additional file 3: Figure S2B). Different settings for the inter-CpG distance (1.5 kb and 2 kb instead of 1 kb) or mismatches (1, 2, 4 and 5 instead of 3) did not appreciably alter the number and length of detected tDMRs, indicating the stability of the algorithm. The DMR finder algorithm was implemented in R statistics and the script is available in Additional file 4. The DMR finder can be used for 450k data (using Illumina CpG identifiers) as well for other types of DNA methylation data (using genomic locations).

### Annotation and enrichment tests

CpGs on the 450k chip were annotated in multiple ways. First, the genome was divided according to five gene-centric regions: the inter-genic region (>10 kb from the nearest TSS), the distal promoter (−10 kb to 1.5 kb from the nearest TSS), the proximal promoter (−1.5 kb to +500 bp from the nearest TSS), the gene body (+500 bp to 3’ end of the gene) and the downstream region (3’ end to +5 kb from 3’ end). Next, CpGs were annotated as non-CGI, CGI, CGI shore or CGI shelf. Genomic locations of CpG islands were obtained from the UCSC browser [41]. CGI shores were defined as 2 kb flanking the CpG island up- and downstream and CGI shelves as 2 kb flanking the CGI shore. Genes displaying tissue-specific expression were obtained from the TiGER database [17]. Alternative transcription/splicing events were downloaded from Ensembl [42-44]. The DNase hypersensitive sites and transcription factor binding sites clustered for multiple cell lines as part of the ENCODE project [20] were downloaded from the UCSC browser. All annotations used in this paper are available from Additional file 8, Additional file 16, Additional file 17 and Additional file 18 as RData objects; these include annotations of genomic features, alternative events, DHSs and transcription factor binding sites. All annotations are based on human genome build 19.

Enrichments, that is, the gene and CpG density centric enrichments, tissue-specific expressed genes, the alternative events, the transcription factor binding sites and the DHSs were calculated using the individual CpG sites within tDMRs. All odds ratios were corrected for background enrichment, which is required because not all CpG sites on the array can become a tDMR as a result of the varying density of the chips. The background odds ratio was determined by identifying tDMR-like regions, that is, regions with an inter-CpG distance smaller than 1 kb with an average length of 5 CpGs per tDMR-like regions (cf. the number of CpGs in identified tDMRs) resulting in ~8 × 10^4^ tDMR-like regions. Reported odds ratios are the calculated odds ratio divided by the background odds ratio. For each enrichment test, we performed 200,001 permutations with 4,500 tDMR-like regions each. Using the resulting empirical distribution, we determined the two-sided *P* value for enrichment.

### Gene ontology term analysis

tDMRs overlapping with an annotation were mapped to the nearest gene using GREAT [45]. Extracted genes were tested for enrichment of GO terms using the GO_BP_FAT table from the DAVID tool [46,47]. To gain further insights regarding the major classes within the significant GO terms, the REVIGO tool was used to cluster and prune GO terms on the basis of *P* values obtained from DAVID, with a medium allowed similarity [48]. Gene region figures were generated using the R package *Gviz*[49] and graphs with the R package *ggplot2*[50].

### Individual variation

To determine individual variation we used liver, subcutaneous fat, omentum and muscle from six autopsy subjects from which we obtained all these tissues. CpGs were mapped to the nearest flanking SNP using the Phase I/II CEU SNPs from the 1000 Genomes project. All SNPs in the probe and CpG SNPs were removed from the data (*n* = 147,963). To determine inter-individual variation we calculated the mean sum of squares for all CpG sites and selected the CpGs with a mean sum of squares >0.025. Correlations between blood and internal tissues were calculated by determining the correlation between all inter-individual comparisons in blood, compared to all inter-individual comparisons in one internal tissue and CpGs with a correlation over 0.8 were selected.

### Data access

The data used in this publication have been deposited in NCBI’s Gene Expression Omnibus [51] and are accessible through GEO Series accession number GSE48472.

## Abbreviations

bp: Base pair; CGI: CpG island; DHS: DNase I hypersensitive sites; DMP: Differentially methylated position; DMR: Differentially methylated region; DTT: Dithiothreitol; EDTA: Ethylenediamine-tetraacetic acid disodium salt dihydrate; HE stain: Hematoxylin and eosin stain; GO: Gene ontology; IO: Internal organ; IT: Internal tissue; kb: Kilobase; OR: Odds ratio; PT: Peripheral tissue; SC: Subcutaneous; SNP: Single nucleotide polymorphism; tDMP: Tissue-specific differentially methylated position; tDMR: Tissue-specific differentially methylated region; TF: Transcription factor; TFBS: Transcription factor binding site; TSS: Transcription start site.

## Competing interests

The authors declare that they have no competing interests.

## Authors’ contributions

BTH, IM, PES, JWJ conceived the experiments. RCS, SDB, RPT, RB and HEDS collected tissue material and performed the laboratory work. RCS and BTH performed the data analysis. JJG, HP, YZ, EWL and EBA provided advice and support for the statistical and bioinformatics analyses. JVMGB provided the autopsy tissues. RCS and BTH drafted the manuscript. All authors read and approved the final manuscript.

## Supplementary Material

Additional file 1: Table S1 Characteristics of subjects and tissues. IT, internal tissue; PT, peripheral tissue. Click here for file

Additional file 2: Figure S1 Quality control figures for both datasets. (A, B) Densities of the quantile normalized beta values. A characteristic bimodal distribution is present as expected. (C, D) The median log2 intensities are high, suggesting the arrays have a decent quality. (E, F) Tissues cluster according to tissue type. SC, subcutaneous. Click here for file

Additional file 3: Figure S2 General characteristics of the data. (A) DNA methylation around the TSS demonstrates a previously observed canonical pattern. (B) Inter-CpG distance versus the absolute difference in beta. Notice that when the inter-CpG distance rises, the difference in DNA methylation also increases with a plateau at 1 kb. (C) Circos representation of the location of the tDMR CpGs in the genome. The three circles from outer to inner are for the internal tissues dataset, the peripheral tissues dataset and the common CpGs between the two, respectively. kb, kilobase; tDMR, tissue-specific differentially methylated region. Click here for file

Additional file 4 **The newly developed DMR finder.** Zip file with the R scripts used to identify DMRs. The *Start file DMRfinder.R* script contains the front end for users and the *DMRfinder.R* the backbone of the algorithm. The algorithm can detect DMRs in 450k data (Illumina IDs/genomic locations) and other types of data (genomic locations). DMR, differentially methylated region. Click here for file

Additional file 5: Table S2 Identified DMRs with associated annotations and intra-individual correlated CpG sites. DMR, differentially methylated region; IT, internal tissue; PT, peripheral tissue. Click here for file

Additional file 6: Figure S3 Heat map of DNA methylation of tDMR CpGs in both datasets. (A) Peripheral tissues. (B) Internal tissues. tDMR, tissue-specific differentially methylated region. Click here for file

Additional file 7: Figure S4 Enrichment and DNA methylation of tDMR CpGs in genes that are expressed in specific tissues. (A) Enrichment of tDMR CpGs in genes that are preferentially expressed in a particular tissue (*x* axis). (B) DNA methylation in the tissues studied of the CpGs that are associated with a gene preferentially expressed in a specific tissue. Notice a drop in methylation in tissue in which it is expressed, while higher methylation is observed in the other tissues. BL, blood; BU, buccal; HA, hair; IT, internal tissue; LI, liver; MU, muscle; OM, omentum; PA, pancreas; PT, peripheral tissue; SA, saliva; SF, subcutaneous fat; SP, spleen; tDMR, tissue-specific differentially methylated region. Click here for file

Additional file 8 **Gene and CpG density centric annotation.** This RData object can be opened with R statistics. It contains a CGI and gene-centric annotation of Illumina 450k CpG sites. CGI, CpG island. Click here for file

Additional file 9: Figure S5 Enrichment of tDMR CpGs in the gene-centric annotation. Blue and pink bars represent enrichment with tDMRs of genomic features in peripheral tissues and internal tissues, respectively, relative to background enrichments. tDMRs are enriched in all genomic features, while depleted in proximal promoters. tDMR, tissue-specific differentially methylated region. Click here for file

Additional file 10: Table S3 Genomic location of tDMRs in both datasets. CGI, CpG island; tDMR, tissue-specific differentially methylated region. Click here for file

Additional file 11: Figure S6 Example of alternative promoter usage. There are more transcription start sites for the DDR1 gene and differential methylation was observed in all proximal promoters of all transcripts. DMR, differentially methylated region; Mb, megabase; SC, subcutaneous; tDMR, tissue-specific differentially methylated region. Click here for file

Additional file 12: Figure S7 DNA methylation of the *SHANK3* gene. A tDMR at a CGI in the *SHANK3* gene body has been reported to regulate alternative transcription [10] and in line with this report we observed differential methylation at the CGIs. CGI, CpG island; Mb, megabase; SC, subcutaneous; tDMR, tissue-specific differentially methylated region. Click here for file

Additional file 13: Figure S8 Enrichment of differentially methylated genes in GO terms. Colours represent major classes of types of GO terms found to be enriched. Notice that tissue-specific genes are mainly enriched in non-CGI features, but also in proximal promoter shelves. CGI, CpG island; GO, gene ontology. Click here for file

Additional file 14: Figure S9 GO term analysis of genes mapping to tDMRs in DHSs, DHS, DNase I hypersensitive site; GO, gene ontology; tDMR, tissue-specific differentially methylated region. Click here for file

Additional file 15: Figure S10 DNA methylation of tDMR CpGs maps to the transcription factor binding sites (above plots). The upper row is the peripheral tissue dataset; bottom row the internal tissue dataset. BL, blood; BU, buccal; HA, hair; LI, liver; MU, muscle; OM, omentum; PA, pancreas; PT, peripheral tissue; SA, saliva; SF, subcutaneous fat; SP, spleen; tDMR, tissue-specific differentially methylated region. Click here for file

Additional file 16 **Annotation of alternative events.** This RData object can be opened with R statistics. It contains an annotation of Illumina 450k CpG sites for Ensembl alternative transcription events. Click here for file

Additional file 17 **Annotation of transcription factor binding sites.** This RData object can be opened with R statistics. It contains an annotation of Illumina 450k CpG sites to ENCODE transcription factor binding sites. Click here for file

Additional file 18 **Annotation of transcription factor binding sites.** This RData object can be opened with R statistics. It contains an annotation of Illumina 450k CpG sites to ENCODE DNA hypersensitive sites. Click here for file
