# Supplementary material for: Identification and systematic annotation of tissue-specific differentially methylated regions using the Illumina 450k array
Source: Epigenetics Chromatin. 2013 Aug 6;6:26. doi: 10.1186/1756-8935-6-26 (PMC3750594; doi:10.1186/1756-8935-6-26)
Supplement: Additional file 1: Table S1 — Characteristics of subjects and tissues. IT, internal tissue; PT, peripheral tissue. [file 1756-8935-6-26-S1.doc]

Supplemental table 1. Characteristics of subjects and tissues

|  | *Gender* | *Age* | *Blood* | *Buccal* | *Hair* | *Saliva* |
| --- | --- | --- | --- | --- | --- | --- |
| PT1 | F | 40 | • | • | • | • |
| PT2 | F | 31 | • | • | • | • |
| PT3 | F | 25 | • | • | • | • |
| PT4 | M | 23 | • | • | • | • |
| PT5 | M | 22 | • | • | • | • |

|  | *Gender* | *Age* | *Blood* | *SC fat* | *Omentum* | *Muscle* | *Liver* | *Spleen* | *Pancreas* |
| --- | --- | --- | --- | --- | --- | --- | --- | --- | --- |
| IT9 | F | 61 | • | • | • | • | • | • |  |
| IT13 | M | 64 | • | • | • | • | • |  | • |
| IT10 | M | 58 | • | • | • | • | • | • |  |
| IT15 | F | 65 | • | • | • | • | • |  | • |
| IT16 | F | 66 | • | • | • | • |  | • | • |
| IT17 | M | 79 | • | • | • | • | • |  | • |
