# Supplementary figures and images for: Identification and systematic annotation of tissue-specific differentially methylated regions using the Illumina 450k array
Source: Epigenetics Chromatin. 2013 Aug 6;6:26. doi: 10.1186/1756-8935-6-26 (PMC3750594; doi:10.1186/1756-8935-6-26)

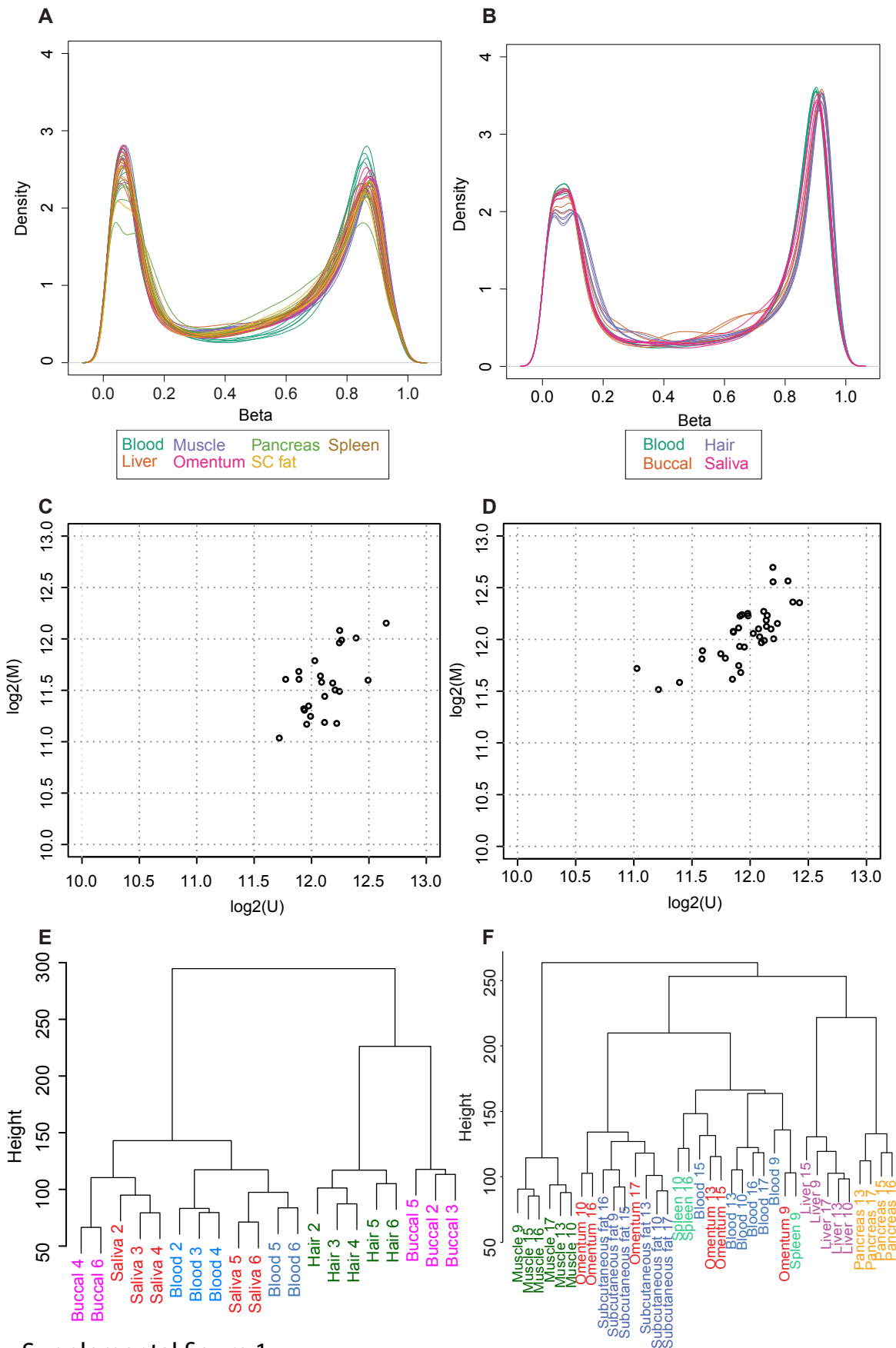

Supplement: Additional file 2: Figure S1 — Quality control figures for both datasets. (A, B) Densities of the quantile normalized beta values. A characteristic bimodal distribution is present as expected. (C, D) The median log2 intensities are high, suggesting the arrays have a decent quality. (E, F) Tissues cluster according to tissue type. SC, subcutaneous. [file 1756-8935-6-26-S2.pdf]

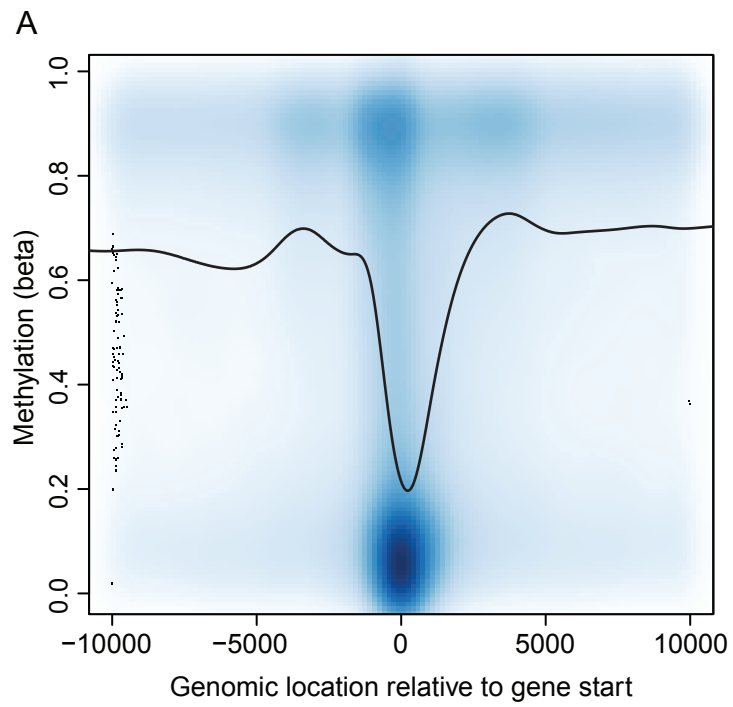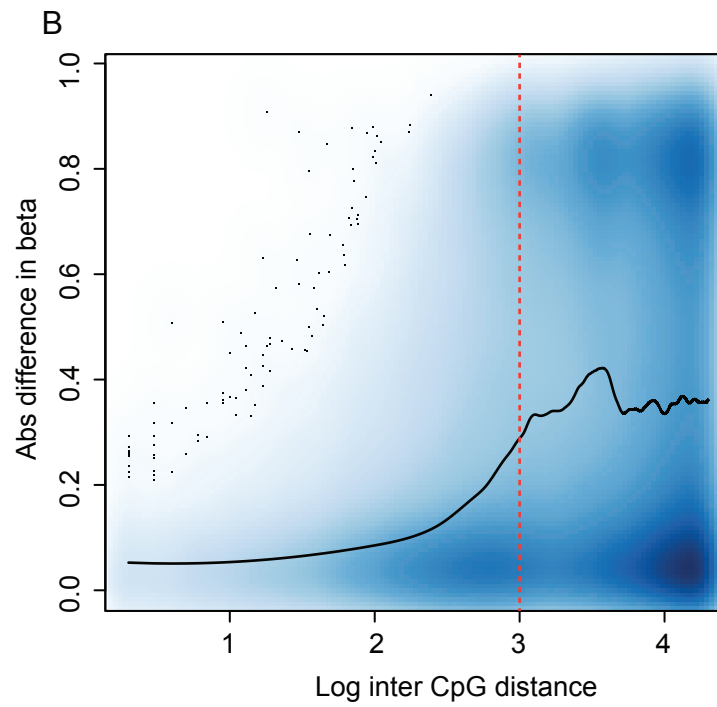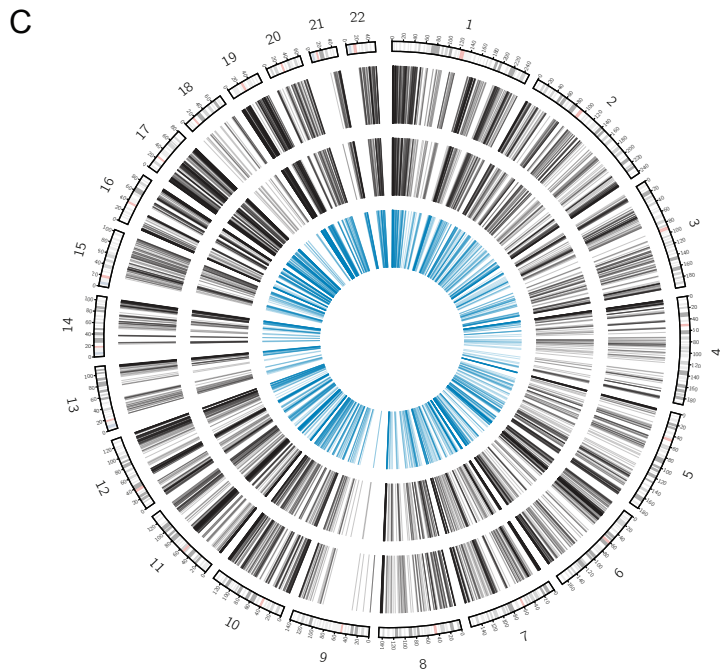

Supplemental figure 2

Supplement: Additional file 3: Figure S2 — General characteristics of the data. (A) DNA methylation around the TSS demonstrates a previously observed canonical pattern. (B) Inter-CpG distance versus the absolute difference in beta. Notice that when the inter-CpG distance rises, the difference in DNA methylation also increases with a plateau at 1 kb. (C) Circos representation of the location of the tDMR CpGs in the genome. The three circles from outer to inner are for the internal tissues dataset, the peripheral tissues dataset and the common CpGs between the two, respectively. kb, kilobase; tDMR, tissue-specific differentially methylated region. [file 1756-8935-6-26-S3.pdf]

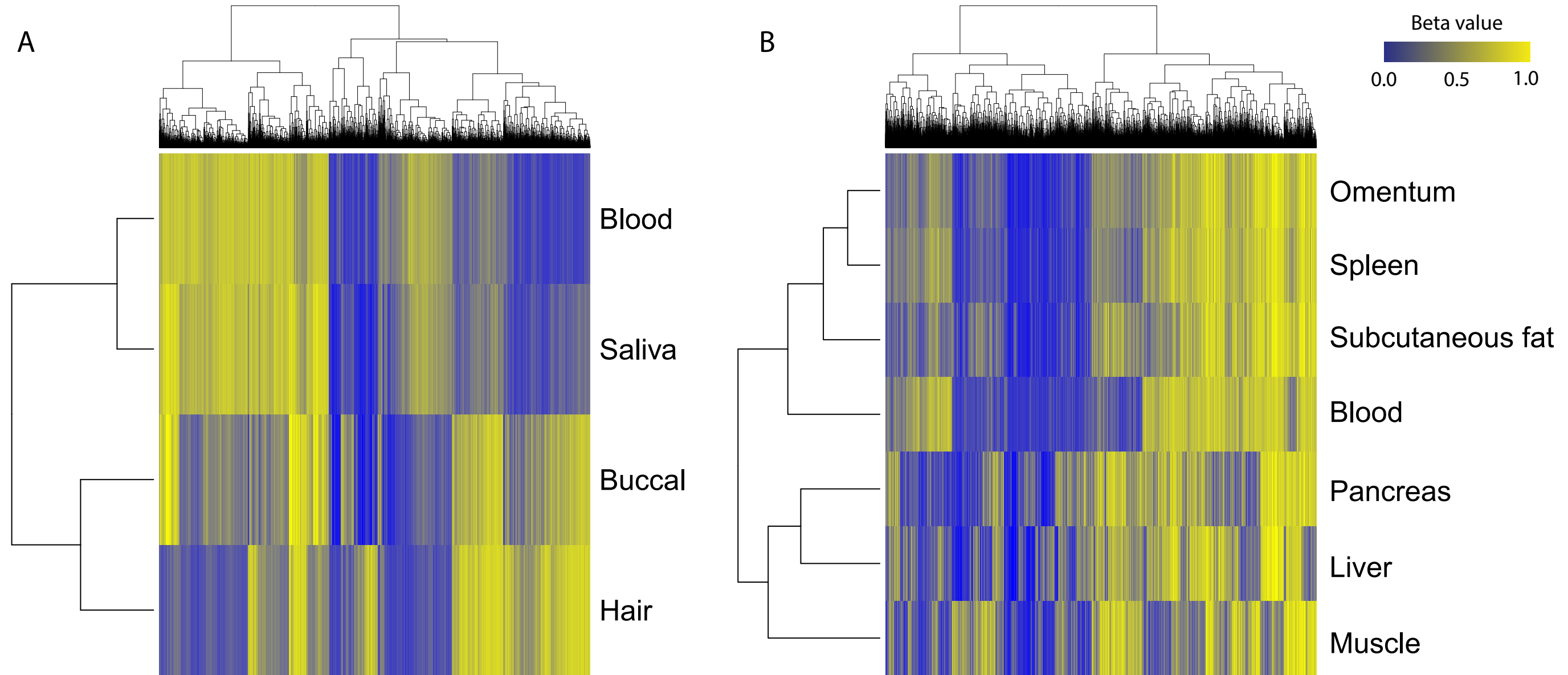

Supplemental figure 3

Supplement: Additional file 6: Figure S3 — Heat map of DNA methylation of tDMR CpGs in both datasets. (A) Peripheral tissues. (B) Internal tissues. tDMR, tissue-specific differentially methylated region. [file 1756-8935-6-26-S6.pdf]

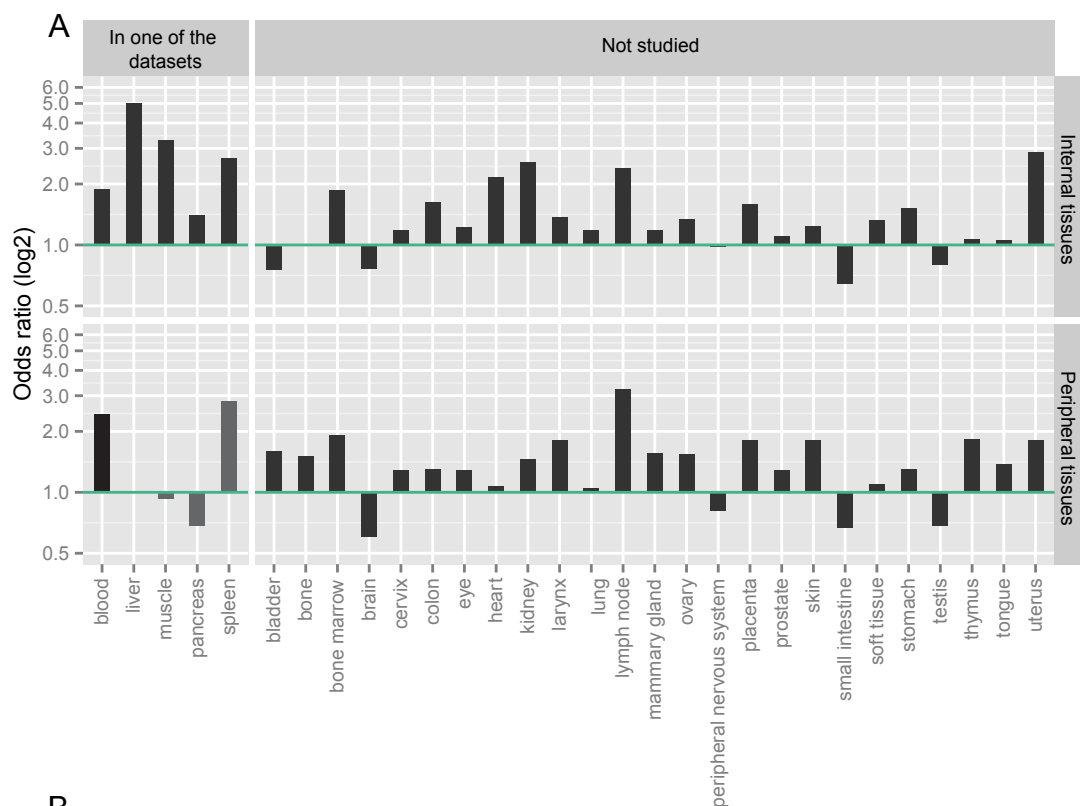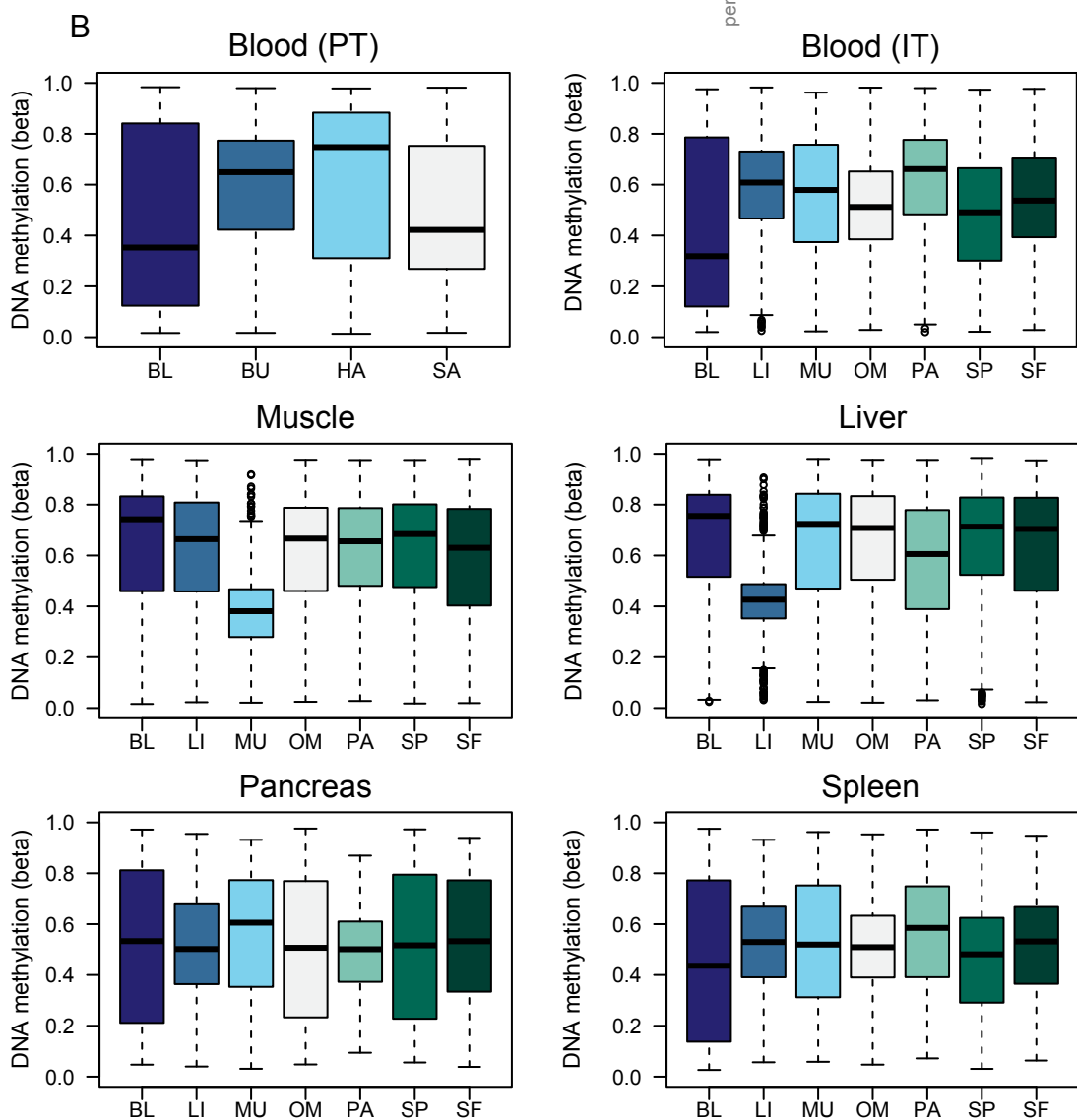

Supplemental figure 4

Supplement: Additional file 7: Figure S4 — Enrichment and DNA methylation of tDMR CpGs in genes that are expressed in specific tissues. (A) Enrichment of tDMR CpGs in genes that are preferentially expressed in a particular tissue (x axis). (B) DNA methylation in the tissues studied of the CpGs that are associated with a gene preferentially expressed in a specific tissue. Notice a drop in methylation in tissue in which it is expressed, while higher methylation is observed in the other tissues. BL, blood; BU, buccal; HA, hair; IT, internal tissue; LI, liver; MU, muscle; OM, omentum; PA, pancreas; PT, peripheral tissue; SA, saliva; SF, subcutaneous fat; SP, spleen; tDMR, tissue-specific differentially methylated region. [file 1756-8935-6-26-S7.pdf]

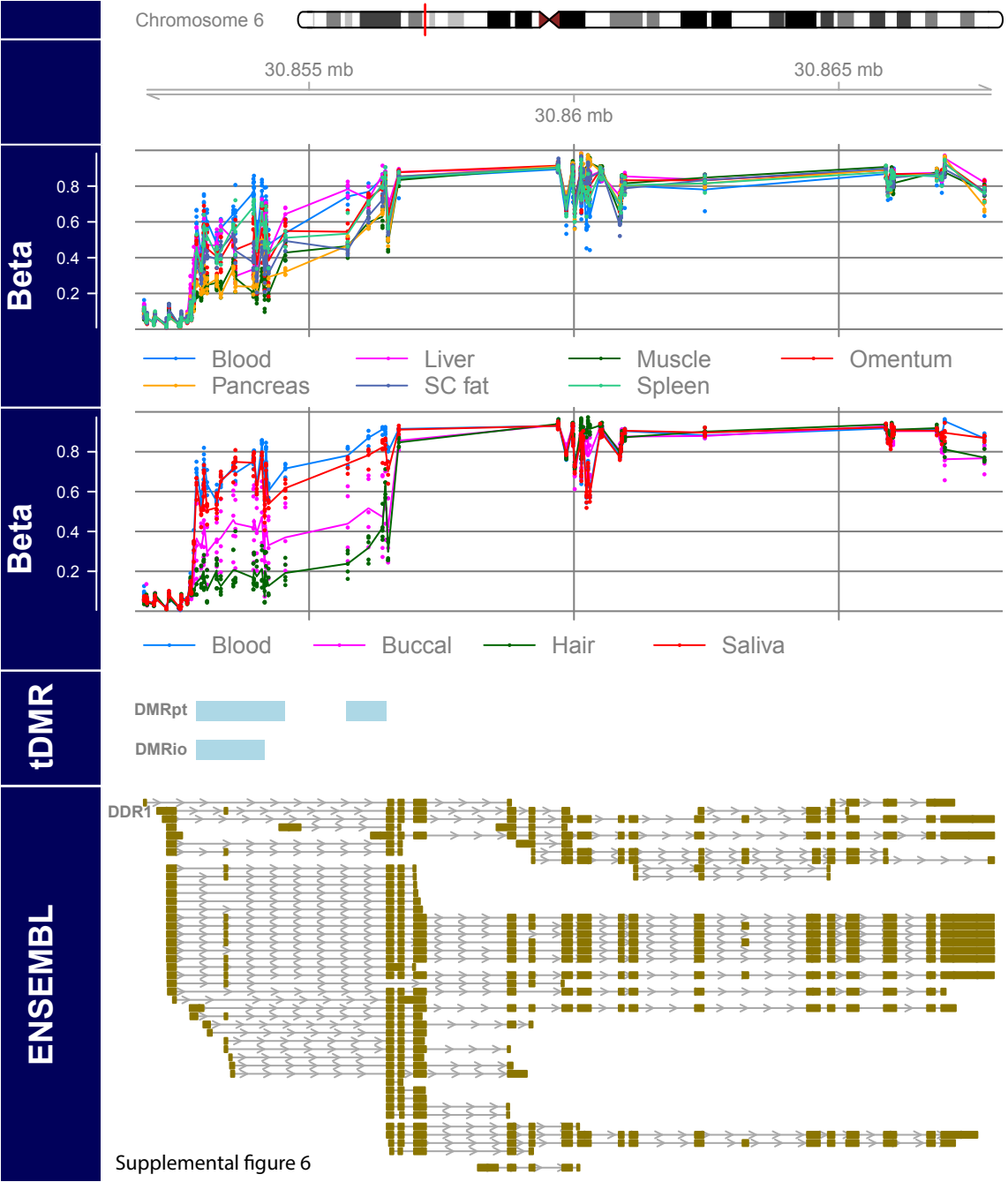

Supplement: Additional file 11: Figure S6 — Example of alternative promoter usage. There are more transcription start sites for the DDR1 gene and differential methylation was observed in all proximal promoters of all transcripts. DMR, differentially methylated region; Mb, megabase; SC, subcutaneous; tDMR, tissue-specific differentially methylated region. [file 1756-8935-6-26-S11.pdf]

Chromosome 22

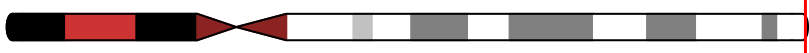

51.12 mb

51.14 mb

51.16 mb

51.13 mb

51.15 mb

Beta

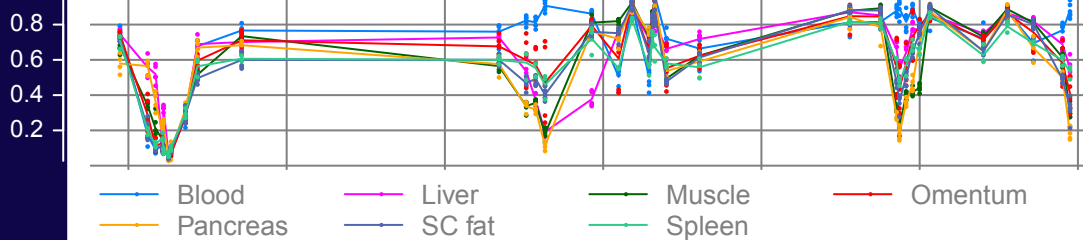

Beta

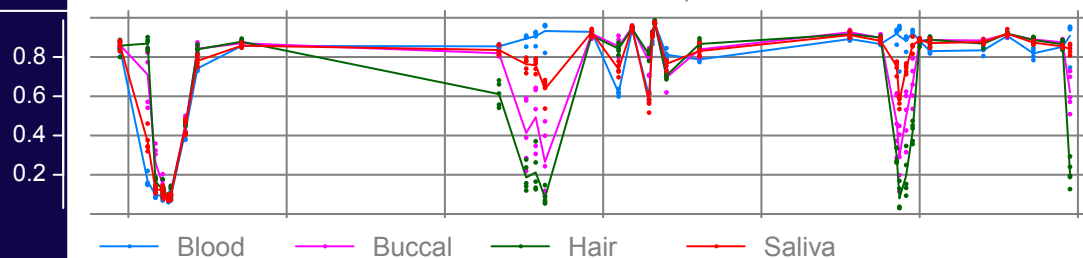

tDMR

DMRpt

DMRio

CGI

ENSEMBL

SHANK3

SHANK3

SHANK3

Supplement: Additional file 12: Figure S7 — DNA methylation of the SHANK3 gene. A tDMR at a CGI in the SHANK3 gene body has been reported to regulate alternative transcription [10] and in line with this report we observed differential methylation at the CGIs. CGI, CpG island; Mb, megabase; SC, subcutaneous; tDMR, tissue-specific differentially methylated region. [file 1756-8935-6-26-S12.pdf]

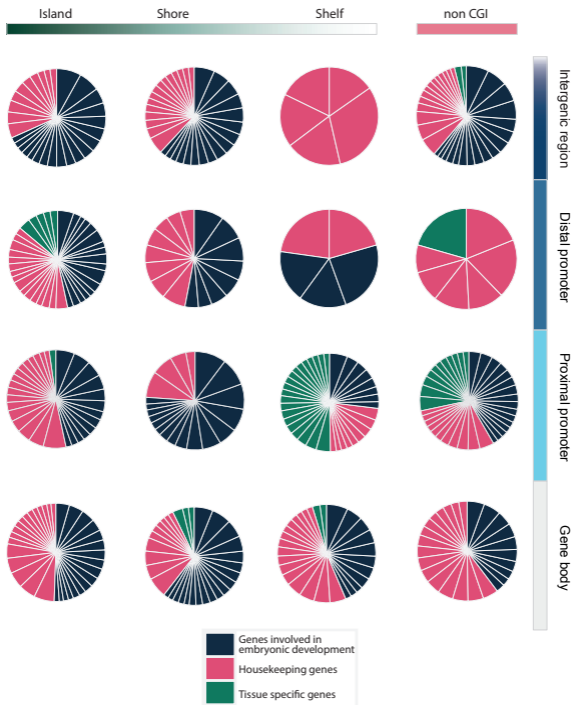

Supplemental figure 8

Supplement: Additional file 13: Figure S8 — Enrichment of differentially methylated genes in GO terms. Colours represent major classes of types of GO terms found to be enriched. Notice that tissue-specific genes are mainly enriched in non-CGI features, but also in proximal promoter shelves. CGI, CpG island; GO, gene ontology. [file 1756-8935-6-26-S13.pdf]

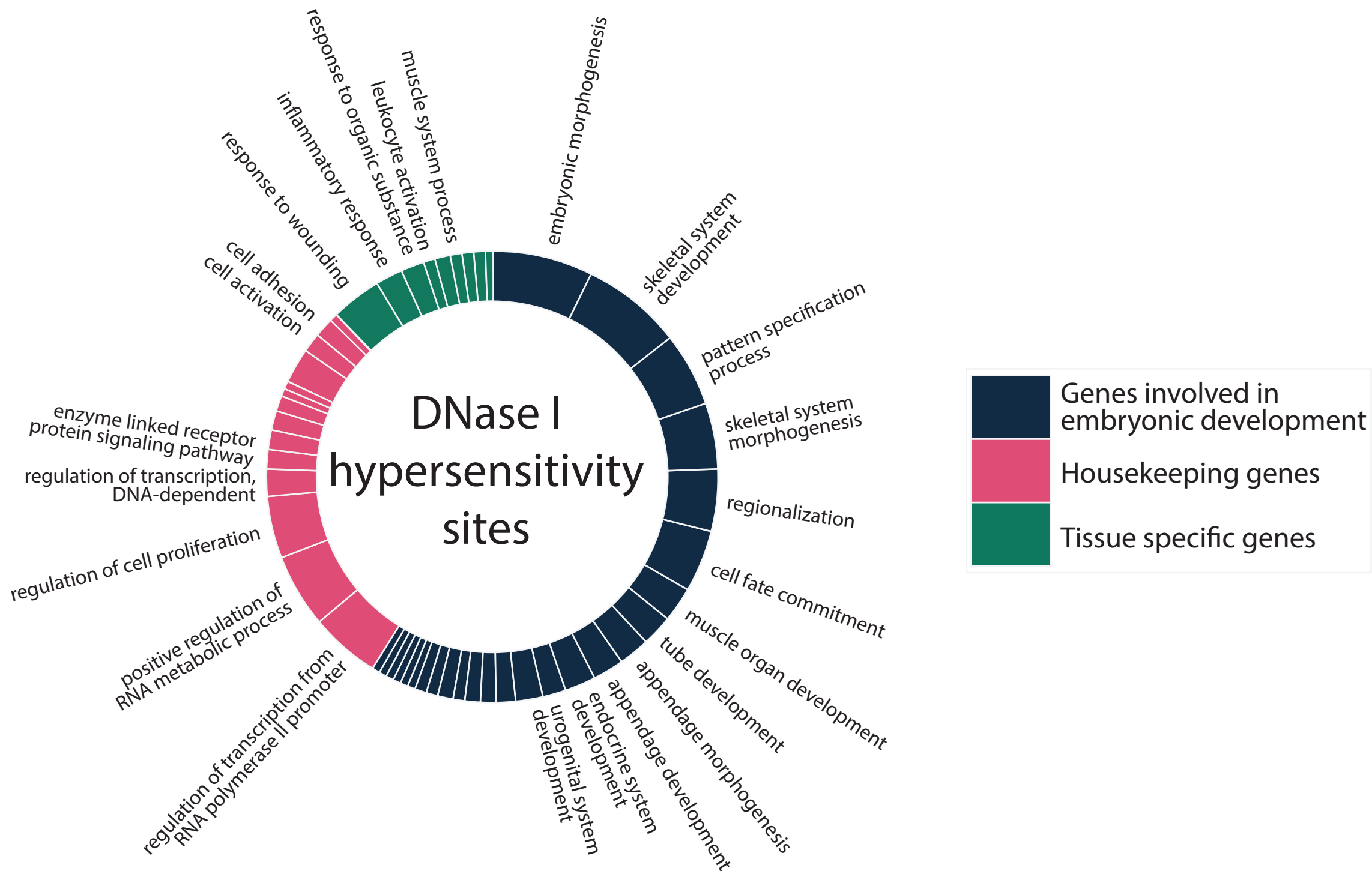

Supplemental figure 9

Supplement: Additional file 14: Figure S9 — GO term analysis of genes mapping to tDMRs in DHSs, DHS, DNase I hypersensitive site; GO, gene ontology; tDMR, tissue-specific differentially methylated region. [file 1756-8935-6-26-S14.pdf]

Beta

BCL11A

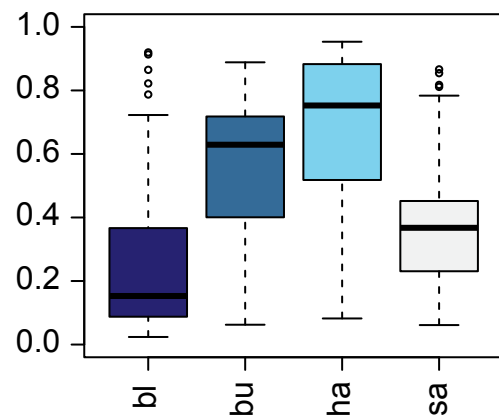

SUZ12

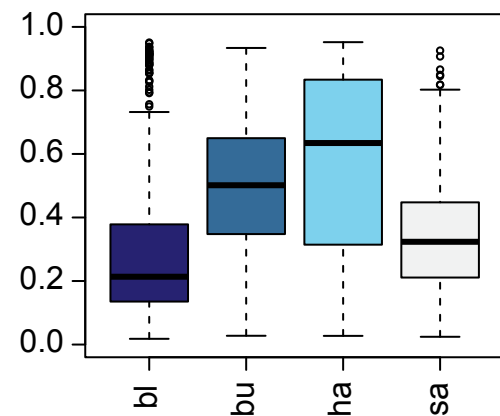

FOXA2

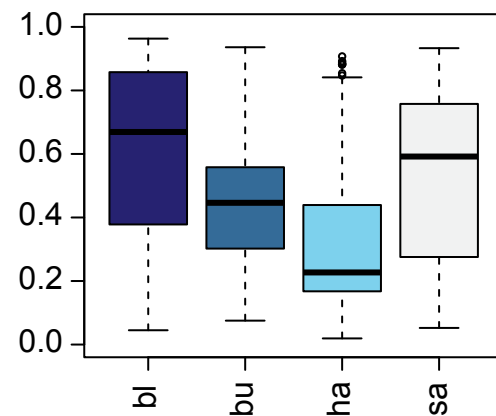

YY1

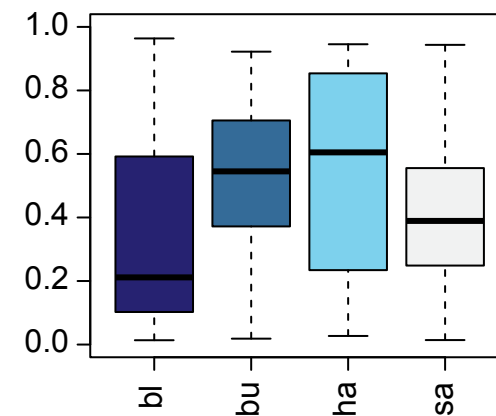

CTCF

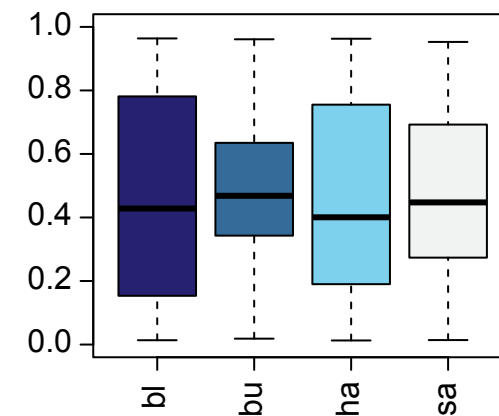

Beta

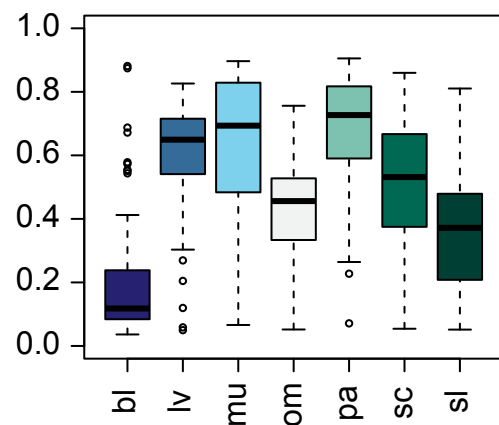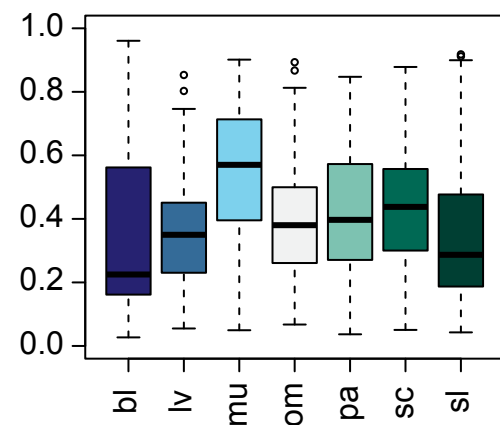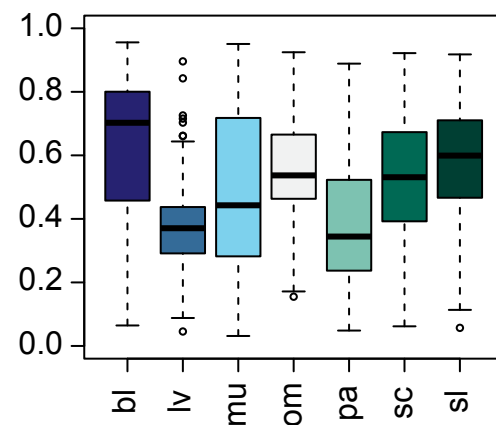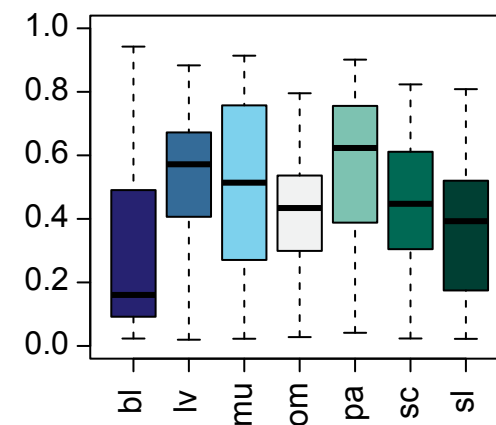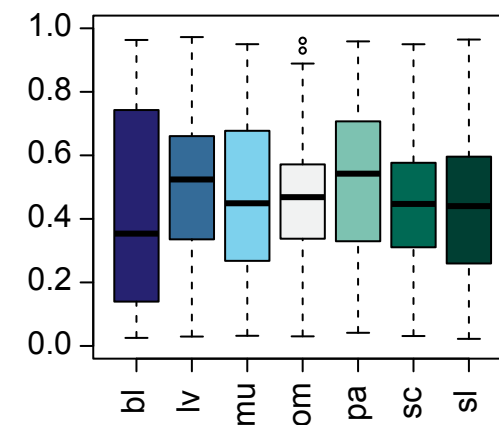

Supplemental figure 10

Supplement: Additional file 15: Figure S10 — DNA methylation of tDMR CpGs maps to the transcription factor binding sites (above plots). The upper row is the peripheral tissue dataset; bottom row the internal tissue dataset. BL, blood; BU, buccal; HA, hair; LI, liver; MU, muscle; OM, omentum; PA, pancreas; PT, peripheral tissue; SA, saliva; SF, subcutaneous fat; SP, spleen; tDMR, tissue-specific differentially methylated region. [file 1756-8935-6-26-S15.pdf]
