# Supplementary material for: Identification and systematic annotation of tissue-specific differentially methylated regions using the Illumina 450k array
Source: Epigenetics Chromatin. 2013 Aug 6;6:26. doi: 10.1186/1756-8935-6-26 (PMC3750594; doi:10.1186/1756-8935-6-26)
Supplement: Additional file 9: Figure S5 — Enrichment of tDMR CpGs in the gene-centric annotation. Blue and pink bars represent enrichment with tDMRs of genomic features in peripheral tissues and internal tissues, respectively, relative to background enrichments. tDMRs are enriched in all genomic features, while depleted in proximal promoters. tDMR, tissue-specific differentially methylated region. [file 1756-8935-6-26-S9.pdf]

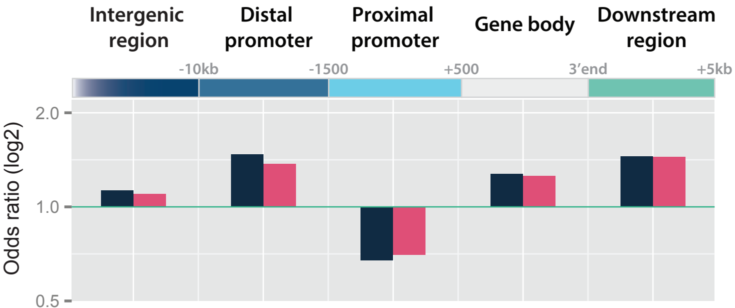

Supplemental figure 5
