# Supplementary material for: Identification and systematic annotation of tissue-specific differentially methylated regions using the Illumina 450k array
Source: Epigenetics Chromatin. 2013 Aug 6;6:26. doi: 10.1186/1756-8935-6-26 (PMC3750594; doi:10.1186/1756-8935-6-26)
Supplement: Additional file 10: Table S3 — Genomic location of tDMRs in both datasets. CGI, CpG island; tDMR, tissue-specific differentially methylated region. [file 1756-8935-6-26-S10.doc]

Supplemental table 2. Genomic location of tDMRs in both data sets

|  | **Peripheral tissues** | | |  | **Internal tissues** | | |
| --- | --- | --- | --- | --- | --- | --- | --- |
| **Name** | **Number of tDMRs** | **Percentage of tDMRs** | **Percentage of CpGs in this feature^1^** |  | **Number of tDMRs** | **Percentage of tDMRs** | **Percentage of CpGs in this feature^1^** |
| **Intergenic CGI** | 130 | 3.7 | 76.6 (18.1) |  | 218 | 4.1 | 77.1 (18.4) |
| **Intergenic Shore** | 82 | 2.3 | 86.3 (17.0) |  | 139 | 2.6 | 83.8 (17.7) |
| **Intergenic Shelf** | 27 | 0.8 | 89.5 (17.2) |  | 34 | 0.6 | 87.9 (15.8) |
| **Non-CGI intergenic** | 260 | 7.4 | 99.6 (3.4) |  | 378 | 7.0 | 99.3 (4.8) |
|  |  |  |  |  |  |  |  |
| **Distal promoter CGI** | 43 | 1.2 | 80.0 (21.4) |  | 91 | 1.7 | 75.4 (20.8) |
| **Distal promoter Shore** | 57 | 1.6 | 83.4 (18.8) |  | 79 | 1.5 | 79.0 (20.3) |
| **Distal promoter Shelf** | 18 | 0.5 | 78.8 (24.2) |  | 18 | 0.3 | 84.2 (18.3) |
| **Non-CGI Distal promoter** | 32 | 0.9 | 96.9 (10.7) |  | 52 | 1.0 | 94.1 (13.6) |
|  |  |  |  |  |  |  |  |
| **Proximal promoter CGI** | 168 | 4.8 | 77.0 (18.7) |  | 313 | 5.8 | 74.8 (18.8) |
| **Proximal promoter Shore** | 417 | 11.8 | 91.1 (15.0) |  | 591 | 11.0 | 88.0 (17.0) |
| **Proximal promoter Shelf** | 96 | 2.7 | 88.0 (16.8) |  | 129 | 2.4 | 85.9 (16.9) |
| **Non-CGI Proximal promoter** | 781 | 22.1 | 97.3 (8.2) |  | 1100 | 20.4 | 97.0 (8.6) |
|  |  |  |  |  |  |  |  |
| **Gene body CGI** | 465 | 13.2 | 79.6 (17.8) |  | 801 | 14.9 | 78.2 (18.1) |
| **Gene body Shore** | 327 | 9.3 | 86.1 (17.6) |  | 470 | 8.7 | 82.1 (18.6) |
| **Gene body Shelf** | 105 | 3.0 | 85.5 (19.3) |  | 148 | 2.7 | 86.1 (19.7) |
| **Non-CGI Gene body** | 447 | 12.7 | 98.6 (6.8) |  | 684 | 12.7 | 97.6 (9.8) |
|  |  |  |  |  |  |  |  |
| **Downstream CGI** | 29 | 0.8 | 73.4 (21.3) |  | 60 | 1.1 | 75.5 (19.5) |
| **Downstream Shore** | 23 | 0.7 | 76.9 (21.5) |  | 40 | 0.7 | 70.0 (23.1) |
| **Downstream Shelf** | 8 | 0.2 | 72.1 (21.7) |  | 6 | 0.1 | 84.7 (17.0) |
| **Non-CGI Downstream** | 18 | 0.5 | 94.7 (12.3) |  | 31 | 0.6 | 97.1 (10.1) |

^1^ Mean percentage of CpGs in single tDMRs of the feature that is mainly represented
